# Safety and efficacy outcomes at 1 year after MitraClip therapy for percutaneous mitral valve repair in patients with severe mitral regurgitation: the Egyptian experience

**DOI:** 10.1186/s43044-021-00166-5

**Published:** 2021-05-03

**Authors:** Radwa Abdullah Elbelbesy, Ahmed Mohsen Elsawah, Ahmed Shafie Ammar, Hazem Abdelmohsen Khamis, Islam Elsayed Shehata

**Affiliations:** 1 Department of Cardiology, Faculty of Medicine, Zagazig University, Zagazig, 44519 Egypt; 2 Department of Cardiology, National Heart Institute, Giza, Egypt

**Keywords:** Mitral Clip, Mitral surgery, Mitral incompetence, Percutaneous repair, Trans-esophageal echo

## Abstract

**Background:**

Our aim was to assess safety and efficacy outcomes at 1 year after MitraClip for percutaneous mitral valve repair in patients with severe mitral regurgitation.

Twenty consecutive patients with significant MR (GIII or GIV) were selected according to the AHA/ACC guidelines from June 2016 to June 2019 and underwent percutaneous edge-to-edge mitral valve repair using MitraClip with a whole 1 year follow-up following the procedure. The primary acute safety endpoint was a 30-day freedom from any of the major adverse events (MAEs) or rehospitalization for heart failure. The primary efficacy endpoint was acute procedural success defined as clip implant with an improvement of MR to ≤ grade II, based on current guidelines, NYHA class, ejection fraction, and the left atrium size during follow-up.

**Results:**

Mean age of the studied population was 66.8 ± 10 years and about 85% were males. All patients presented with NYHA > 2. EuroSCORE ranged between 7 and 15. Patients varied regarding their HAS-BLED score. None of them experienced MAEs at 30 days. Patients showed significant improvement of NHYA functional class, and all echocardiographic measurements such as left ventricular end systolic diameter, left ventricular end diastolic diameter, left ventricular ejection fraction, left atrium volume index and MR grade. They also showed significant improvement of right-side heart failure manifestations (lower limb edema, S3 gallop, neck veins congestion), and laboratory value (the mean Hb levels significantly increased from 11.96 ± 1.57 to 12.97 ± 1.36, while the median CRP significantly decreased from 7 (3-9) to 2 (1-3). As well, the median Pro-BNP significantly decreased from 89.5 (73-380) to 66.5 (53.5-151) following MV clipping. During the whole follow-up period, there was dramatic improvement in the NHYA functional class, echocardiographic assessment including left ventricular ejection fraction, and mitral regurge grade. During follow-up, four patients (20%) developed complications. There was no statistical difference between patients who developed complications and those who did not regarding their age (75.25 ± 12.42 versus 64.63 ± 9.21, respectively), BSA (1.69 ± 0.11 versus 1.79 ± 0.22, respectively), gender (75% versus 87.5% males respectively), MR etiology (75% versus 50% ischemic, 25% versus 50% non-ischemic), or NYHA pre- or post-mitral clipping. However, the median EuroSCORE was significantly higher in the complicated group (13, IQR= 11.5-14.5) than the non-complicated group (9.5, IQR=8.5-11.5).

**Conclusion:**

Percutaneous usage of MitraClip for mitral valve repair showed favorable reliability and better clinical outcomes.

**Trial registration:**

ZU-IRB#2481-17-2-2016 Registered 17 February 2016, email: IRB_123@medicine.zu.edu.eg

## Background

Mitral incompetence is the second clinically relevant adult valvular disease with an estimated yearly incidence of 2-3% [1].

Despite optimization of medical therapy and device therapy in those with heart failure candidate for resynchronization, some patients are still non responders and severe MR can be still or even go from bad to worse in which MV surgery can be an optimal choice [2]. However a large percentage of patients have a high perioperative risk for various reasons [3, 4] what makes a less invasive percutaneous treatment an excellent alternative, yet most of these techniques are still under evaluation with no solid data regarding safety [5]. The MitraClip (Abbott Vascular, Santa Clara CA, USA) was the very first system for MV repair and has been used in over 6000 patients since 2003. The FDA already approved MitraClip device for symptomatic MR (MR≥3+) caused by primary MR in patients with proved high surgical risk [6].

The MitraClip device is a percutaneous edge-to-edge attachment system resembling surgery. The technique creates a tissue bridge between the anterior and posterior leaflets by means of one clip deployed through trans-septal approach. Early trials presumed that MitraClip is feasible, reliable, and safe with about 60% discharged with trivial or mild residual MR [7].

The first randomized controlled study concerned with percutaneous MV repair, the EVEREST II trial (Endovascular Valve Edge-to-Edge Repair Study), compared MitraClip with surgical MV repair and demonstrated the superior safety of the MitraClip repair with comparable clinical results [8]. Moreover, until the EVEREST II trial, the evidence base was remarkably lacking in high-level clinical trials studying the outcomes of any intervention for severe MR, surgical or percutaneous [9]. So, this study aimed to assess the long-term reliability and efficacy of MitraClip for percutaneous mitral valve repair in patients with severe mitral regurgitation.

## Methods

### Study site

Our study was conducted at our Hospital’s Cardiology department. The protocol was approved by the University Institutional Review Board (IRB) which confirmed that all methods were performed in accordance with the relevant guidelines and regulations. Informed written consent was obtained from all participants.

### Time frame

Patients were recruited for 36 months from June 2016 to June 2019.

### Study population

The study group comprised 20 consecutive patients with moderate (III+) to severe (IV+) functional or degenerative MR with symptoms as well as asymptomatic patients with compromised left ventricle (LV) function who fulfilled the inclusion criteria and followed up over a whole year.

### Study design

A single center prospective cohort study.

### Sample size estimation

From June 2016 to June 2019, a convenience sample of 20 patients who fulfilled the eligibility criteria was treated with the MitraClip™ device.

### Eligibility criteria

#### Inclusion criteria

Patients with moderate (III+) to severe (IV+) functional or degenerative MR with symptoms as well as asymptomatic patients with compromised left ventricle (LV) function (ejection fraction < 60% or end-systolic dimension > 45 mm) who had high risk for surgery (logistic EuroSCORE > 20%).

#### Exclusion criteria

Patients with any of the following: recent myocardial infarction in the past 12 weeks prior to Mitraclipping or MV surgery, MV area <4 cm^2^, severe annular calcification, hypertrophic cardiomyopathy and SAM (systolic anterior motion) of any cause, proved intra cardiac mass, thrombus or vegetations, patients who are not hemodynamically stable, patients with active endocarditis, rheumatic heart disease, recent cerebrovascular insult whether stroke or TIA (<6 month), flail mitral valve leaflets (flail width > 15 mm, flail gap >10 mm), any anatomical leaflet abnormality that make a MitraClip impossible to fit and be reliably positioned, as well as any contraindication for trans-esophageal echocardiography (TEE).

### Study endpoints

The primary acute safety endpoint was a 30-day freedom from any of the major adverse events (MAEs) that included the following: cardiovascular death, myocardial infarction, transfusion of > 2 units of blood, kidney failure, non-elective cardiac surgery for adverse events, and mechanical ventilation for more than 48 h, deep wound infection or septicemia or rehospitalization for heart failure. The primary efficacy endpoint was acute procedural success defined as clip implant with an improvement of MR to ≤ grade II, based on current guidelines, NYHA class, ejection fraction, and the left atrium size during follow-up.

Grade III MR was assigned as recommended by the American Society of Echocardiography (ASE) based on a validated integrative method and the consensus of two expert observers. In case of disagreement, the final decision was made by consensus after opinion of the third observer. As regurgitant orifice area and vena contracta width have not been validated for a double-orifice valve, these parameters were not involved among methods to evaluate the severity of MR.

### Tools and instruments

Conventional echocardiography (TTE or TEE) and the MitraClip system.

### Study methodology

For 20 consecutive patients who fulfilled the eligibility criteria, complete history taking, general examination, and vital data assessment were carried out. Local examination of the heart for cardiomegaly, pulsations, thrills, heart sounds, and murmur was conducted. The conventional transthoracic echocardiography was done and measures were indexed to mean body surface area.

**Fig. 1:**
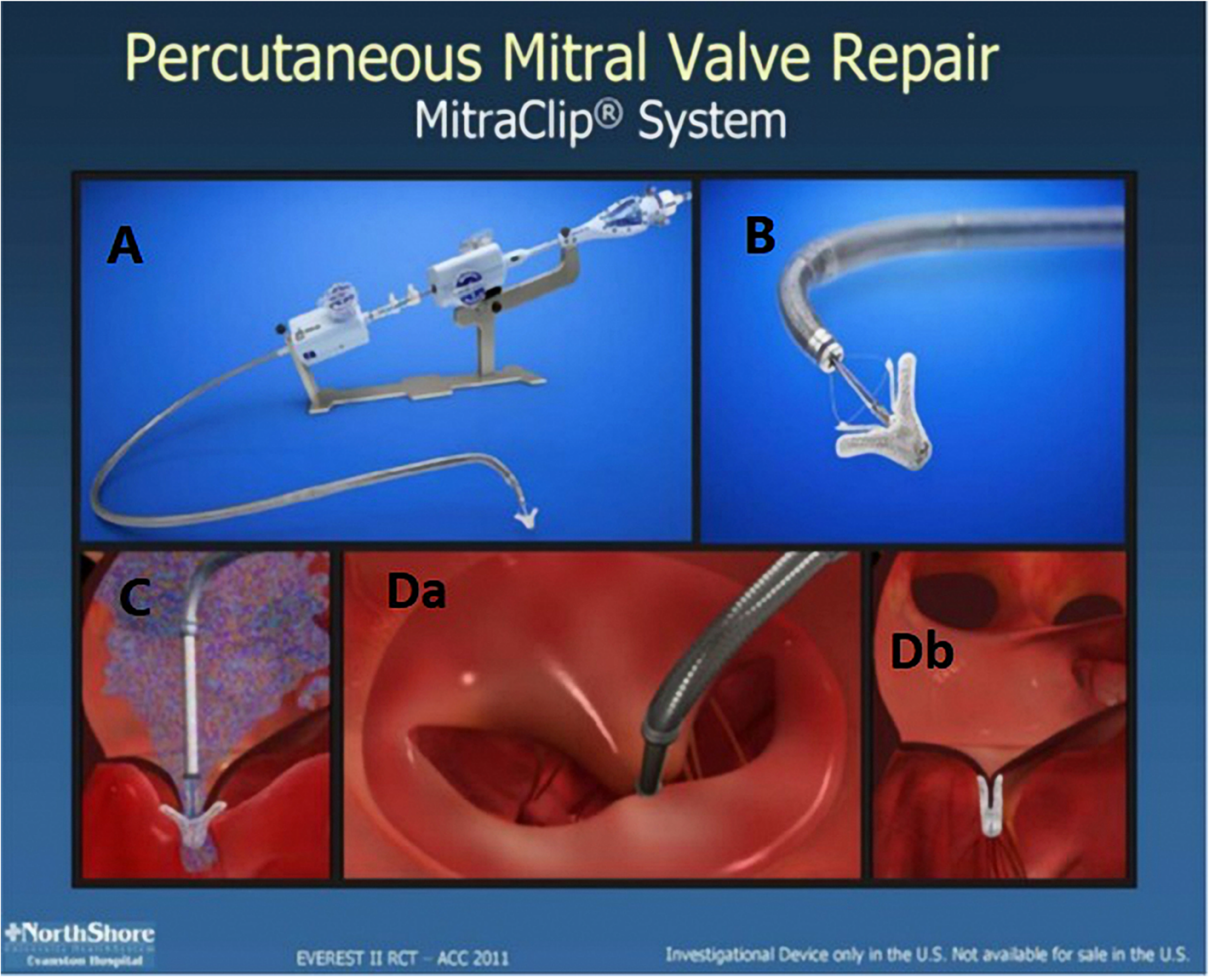
The MitraClip™ Percutaneous Edge-to-Edge Repair System. The MitraClip™ delivery system (Abbott Vascular, Abbott Park, IL) showing a steerable guide catheter, a clip delivery system, and the MitraClip™ device (**a**). The MitraClip™ device is a polyester-covered cobalt-chromium implant with two arms, while the *U* shaped grippers are placed in the inner portion of the clip that helps with leaflet fixation (**b**). The clip delivery system exits through a guide catheter, to grasp the mitral valve leaflet at the site of MR (**c**). When the clip is closed, the leaflet tissue is secured by the clip arms on ventricular side and by the grippers on the atrial side, creating a double-orifice valve (**d**)

**Fig. 2:**
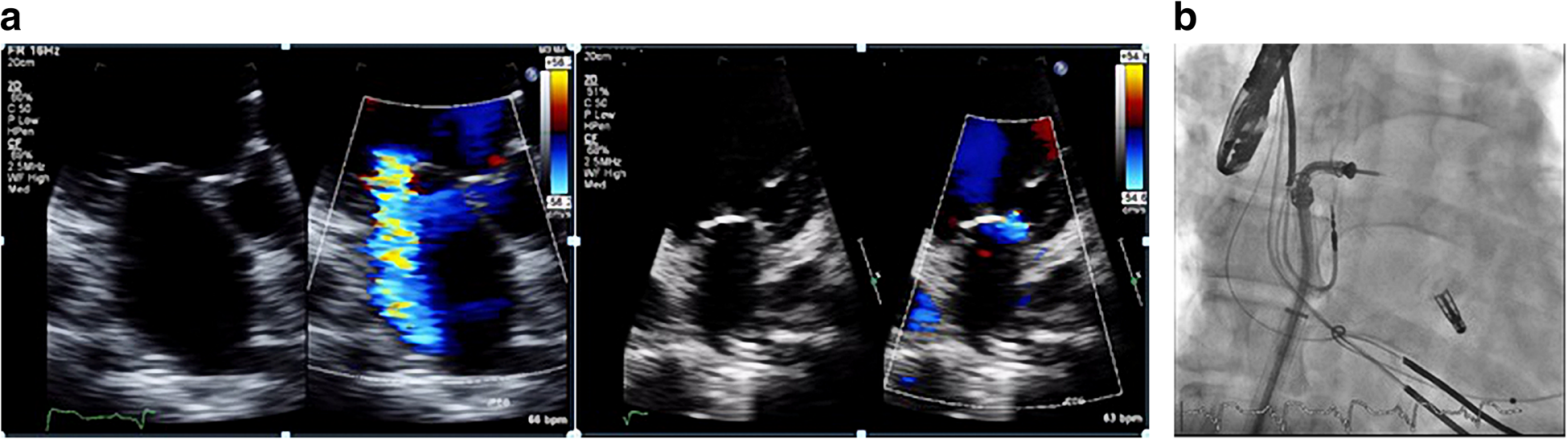
(Case 1) 48-year-old male patient with ischemic MR, NYHA class III, and EuroScore 10. **a** Shows the mitral valve before and after MitraClip deployment. **b** Shows intra-procedure image of MitraClip deployment

Grading of MR severity was carried out according to the American Society of Echocardiography (ASE) guidelines, using quantitative (regurgitant volume and regurgitant fraction) and qualitative (color Doppler and pulmonary venous flow) criteria. These included a regurgitant jet origin associated with the A2 to P2 segments of the MV and, for patients with functional MR, a coaptation length of at least 2 mm, a coaptation depth of no more than 11 mm, and for patients with flail leaflet, a flail gap < 10 mm and a flail width < 15 mm (Figs. 1 and 2) as previously reported by Biner et al. [10].

### Technical steps for percutaneous edge-to-edge mitral valve repair by the MitraClip

- The MitraClip apparatus included a MitraClip device, a 24-F steerable guide catheter, and a delivery system. The clip was pre-assembled to the tip of the disposable delivery catheter. Opening, closing, locking, and detaching the clip were all controlled by the delivery catheter handle. The procedure to be done under general anesthesia and TEE guidance. The right femoral vein was cannulated with an 8-F introducer sheath; then, exchanged with an 8-F Mullins sheath (S. Jude Medical, Minnesota, USA) over a 0.32 guide wire, and a trans-septal puncture was performed using a Brockenbrough needle assisted by TEE. The puncture, targeted at postero-superior part of the interatrial septum to ensure enough room in the left atrium for safe and optimal orientation of the steerable distal part of the clip delivery sheath. Upon entering the left atrium with the 8-F sheath, the left upper pulmonary vein was cannulated using a 260-cm Amplatz Super stiff guide wire.
- After giving of 100 IU/kg of unfractionated heparin, the 24-F steerable guiding catheter was introduced in the left atrium; the dilator was carefully and slowly regained to avoid vacuum air bubbles. The clip delivery sheath was then advanced in the left atrium, and the distal steerable part was positioned properly to be perpendicular and central with respect to the MV leaflets coaptation line. The correct trajectory of the clip and the perpendicularity of the two arms with respect to the mitral coaptation line were checked using three different echocardiographic windows (three chamber, dual chamber, and trans-gastric short axis view).
- As the device is properly aligned, the clip with opened arms was advanced into the LV and under TEE guidance the arms grasp the leaflets. When a double orifice had been created and the echocardiography confirmed acceptable reduction in the incompetence degree together without any significant diastolic gradient across the MV; grasping of both leaflets, there were two options: if the position was suboptimal, the clip was reopened and repositioned; if the result was good and the grasp was stable, the clip arms were closed, locked, and detached, the steerable guiding catheter and the delivery sheath were withdrawn, removing the guiding catheter and venous femoral sheath (Fig. 1).

### Follow-up

**Fig. 3:**
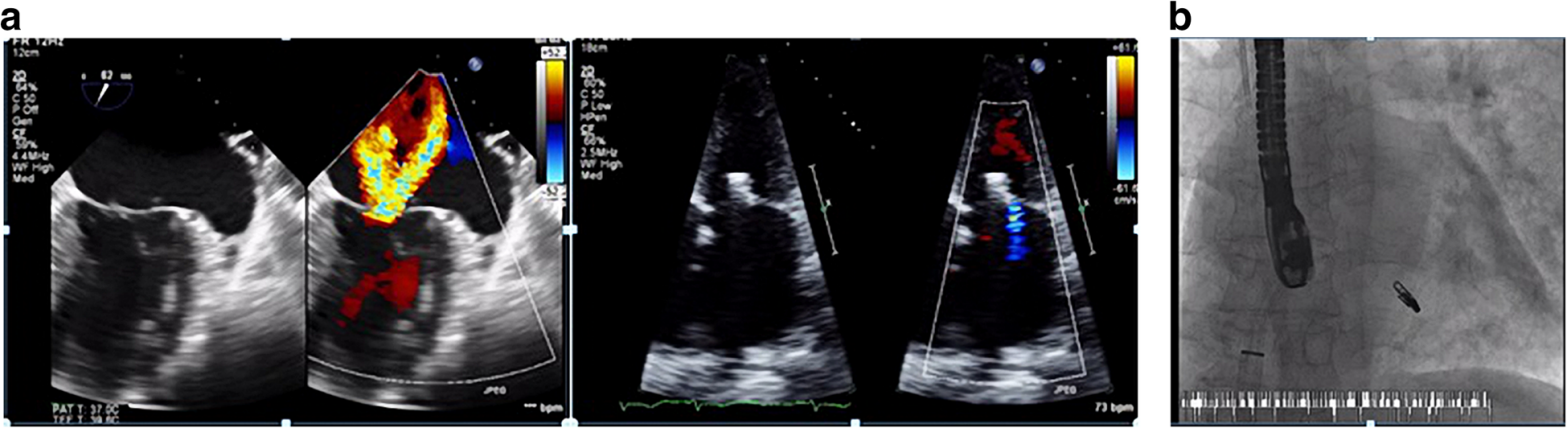
(Case 2) 69-year-old female patient with functional MR, AF, NYHA class III, and EuroScore 9. **a** The mitral valve before and after MitraClip deployment. **b** Intra-procedure image of MitraClip deployment

*Intra procedural: Acute procedural success was highlighted mainly by improving the degree of MR to less than ≤ II without any complications (Figs. 2 and 3).

*1st, 6th, and 12th month: Follow up at 1st, 6th, and 12th months done to ascertain complete freedom from MAEs, to assess NYHA class, echocardiographic parameters, left atrium dimensions and right-side heart failure manifestations.

*We assessed the patients for the development of any complications. Four (20%) developed complications. The complicated and the non-complicated groups were compared.

### Statistical methods

All data were analyzed using the SPSS software version 22 (SPSS, Inc. Chicago, IL, USA). The statistical analysis was conducted following the principles as specified in International Council for Harmonization (ICH) Topic E9 (ICH1998). Results were presented as mean value ± SD for continuous variables and as frequency (%) for categorical variables. Data were tested for normality using the Kolmogorov-Smirnov test. Means were compared using Student *t* test or Mann-Whitney test. Categorical data were compared using the chi-squared test. Comparison of the follow-up data at the studied time points was done using one-way repeated measures ANOVA for quantitative data with parametric distribution, while data with non-parametric distribution were analyzed by Freidman test. Both tests were followed by post hoc analysis for pairwise comparison when they had significant results. The confidence interval was set to 95% and the margin of error accepted was set to 5%. For all statistical analyses, a *P* value of less than or equal to 0.05 was considered to indicate a significant difference.

## Results

### Baseline clinical characteristics

Age of the studied patients was 66.8±10.5 years old (range, 46-84 years old). The majority (85%) of the study participants were males, and the body surface area was 1.77±0.2 m^2^. Mitral regurgitation assessment showed variable etiologies. Ischemic, functional, and flail MR were found in 55%, 30%, and 15% of patients, respectively. All patients presented with NYHA > 2. NYHA III and NYHA IV were found in 75% and 25% of patients, respectively. EuroSCORE ranged between 7 and 15. Patients varied for their HAS-BLED score with median value of 1.0 (range, 0-4). Coronary angiography showed that 35% of patients had previous PCI, while 45% had normal coronary anatomy (Table 1).

**Table 1:** Participants’ demographics

Variables		Total ***N*** = 20
Age (years)	Mean ± SD	66.8 ± 10.5
	Range	46-84
Gender	Female	3 (15%)
	Male	17 (85%)
BSA (m^2^)	Mean ± SD	1.77 ± 0.2
	Range	1.55-2.3
MR etiology	Ischemic	11 (55%)
	Functional	6 (30%)
	Flail	3 (15%)
NYHA class pre	I	0 (0%)
	II	0 (0%)
	III	15 (75%)
	IV	5 (25%)
EuroSCORE	Median (IQR)	11 (9-12.5)
	Range	7-15
HAS-BLED score	Median (IQR)	1 (1-2)
	Range	0-4
Coronary angiography and intervention	Normal CAG	9 (45%)
	TVD for medical treatment	2 (10%)
	LM	1 (5%)
	PCI	7 (35%)
	RCA	5 (25%)
	LAD	4 (20%)
	Non-significant CAD	2 (10%)
	LCX	4 (20%)

*N* Number, *SD* Standard deviation, *IQR* Interquartile range, *BSA* Body surface area, *MR* Mitral regurgitation, *NYHA* New York Heart Association, *EuroSCORE* European System for Cardiac Operative Risk Evaluation, *HAS-BLED* Hypertension, abnormal liver/renal function, stroke history, bleeding history or predisposition, labile INR, elderly, drug/alcohol usage, *CAG* Coronary angiography, *TVD* Triple-vessel disease, *LM* Left main coronary artery, *PCI* Percutaneous coronary intervention, *RCA* Right coronary artery, *LAD* Left anterior descending, *CAD* Coronary artery disease, *LCX* Left circumflex

### Procedure characteristics

Patients showed significant improvement for their NYHA class following MV clipping. NYHA class III significantly reduced from 75 to 0.0% and NYHA class IV also significantly reduced from 25 to 0.0% as shown in Table 2. They had significant improvements regarding echocardiographic assessments: LVES, 49.8±6.3 to 45.1±5.4; LVED, 64.6±5.2 to 61.2±5.0; and LVEF, 30±9.2 to 36.1±7.9. Left atrium dimensions significantly improved with improvement of all parameters of MR. LA volume index, 47.8±2.6 to 43.6±2.7; MR grade, 4±0.2 to 1.8±0.5; vena contracta width, 11.5±1.9 to 5.9±1.7; jet area 13.2±1.6 to 6.9±1.9; regurgitant fraction, 59.4±5.4 to 41±7.5; regurgitant volume, 67.8±6.9 to 44.4±13.6; PISA radius, 12.3±1.9 to 6.2±2; EROA, 0.7±0.1 to 0.3±0.1; ESPAP, 63.1±7.2 to 46.2±6.9; and TAPSE, 2±0.5 to 2.3±0.4 (*P*=0.000*).

**Table 2:** Paired comparisons between pre- and post-mitral clipping measurements

Variables		Pre	Post	***P*** value
**NYHA class**	I	0 (0%)	14 (70%)	0.000*
	II	0 (0%)	6 (30%)	
	III	15 (75%)	0 (0%)	
	IV	5 (25%)	0 (0%)	
**LV EDD**	Mean± SD	64.6 ± 5.23	61.15 ± 5	0.000*
	Range	57-74	54-71	
**LV ESD**	Mean± SD	49.75 ± 6.32	45.05 ± 5.36	0.000*
	Range	38-62	34-57	
**EF**	Mean± SD	29.55 ± 9.23	36.1 ± 7.91	0.000*
	Range	15-50	25-55	
**LA volume index (ml/m**^**2**^**)**	Mean± SD	47.8 ± 2.63	43.6 ± 2.7	0.000*
	Range	44-53	38-48	
**MR grade**	Mean±SD	3.95 ± 0.22	1.8 ± 0.52	0.000*
	Range	3-4	1-3	
	1	0 (0%)	5 (25%)	
	2	0 (0%)	14 (70%)	
	3	1 (5%)	1 (5%)	
	4	19 (95%)	0 (0%)	
**Vena contracta width**	Mean± SD	11.5 ± 1.91	5.85 ± 1.66	0.000*
	Range	7-14	4-11	
**Jet area**	Mean± SD	13.2 ± 1.64	6.85 ± 1.9	0.000*
	Range	11-16	4-11	
**Regurgitant fraction**	Mean± SD	59.4 ± 5.41	40.95 ± 7.48	0.000*
	Range	50-68	26-59	
**Regurgitant volume (ml/beat)**	Mean± SD	67.8 ± 6.93	44.4 ± 13.57	0.000*
	Range	60-85	20-66	
**PISA Radius (mm)**	Mean± SD	12.25 ± 1.92	6.2 ± 1.96	0.000*
	Range	10-16	3-10	
**EROA (cm**^**2**^**)**	Mean± SD	0.65 ± 0.11	0.32 ± 0.12	0.000*
	Range	0.47-0.85	0.14-0.65	
**ESPAP**	Mean± SD	63.1 ± 7.24	46.2 ± 6.89	0.000*
	Range	50-78	37-60	
**TAPSE**	Mean± SD	2.02 ± 0.46	2.29 ± 0.35	0.000*
	Range	1.3-2.6	1.6-2.7	

*SD* Standard deviation, *NYHA* New York Heart Association, *LVEDD* Left ventricle end-diastolic dimensions, *LVESD* Left ventricle end-systolic dimensions, *EF* Ejection fraction, *LA* Left atrium, *MR* Mitral regurgitation, *PISA* Proximal isovelocity surface area, *EROA* Effective regurgitant orifice area, *ESPAP* Estimated systolic pulmonary artery pressure, *TAPSE* Tricuspid annular plane systolic excursion

^*^Significant

Table 3 shows significant improvements in patients’ symptoms following MV clipping. Lower limb edema decreased from 85 to 55% and congested neck veins were less detected from 70 to 20% of patients. Additional heart sounds disappeared. However, atrial fibrillation (AF) status did not change significantly.

**Table 3:** Paired comparisons pre- and post-mitral clipping for right-side heart failure manifestation

Variables		Pre		Post		***P*** value
		***N***	%	***N***	%	
**Lower limb edema**	No edema	3	15%	9	45%	0.011*
	Ankle	7	35%	10	50%	
	Chin	6	30%	1	5%	
	Thigh	4	20%	0	0%	
**AF**	Absent	11	55%	14	70%	0.327
	Exist	9	45%	6	30%	
**S3 gallop**	No	14	70%	20	100%	0.008*
	Yes	6	30%	0	0%	
**Neck veins congestion**	Not congested	6	30%	16	80%	0.004*
	Congested in 45 degrees	11	55%	4	20%	
	Congested in upright position	3	15%	0	0%	

*N* Number, *AF* Atrial fibrillation

^*^Significant

Table 4 shows that the mean Hb levels significantly increased from 11.96 ± 1.57 to 12.97 ± 1.36, while the median CRP significantly decreased from 7 (3-9) to 2 (1-3). As well, the median Pro-BNP significantly decreased from 89.5 (73-380) to 66.5 (53.5-151).

**Table 4:** Paired comparison of laboratory values

Variables		Pre	Post	***P*** value
**Hb**	Mean ± SD	11.96 ± 1.57	12.97 ± 1.36	0.000*
	Range	7-14.5	10.8-15.7	
**CRP**	Median (IQR)	7 (3-9)	2 (1-3)	0.000*
	Range	1-12	1-8	
**Pro-BNP**	Median (IQR)	89.5 (73-380)	66.5 (53.5-151)	0.000*
	Range	45-670	34-320	

*SD* Standard deviation, *IQR* Interquartile range, *Hb* Hemoglobin, *CRP* C-reactive protein, *Pro-BNP* Pro b-type natriuretic peptide

^*^Significant

### Clinical outcome and follow up

In the present study, four (20%) patients developed complications. Out of the complicated cases, two (50%) developed bleeding that needed blood transfusion, one (25%) developed ischemic CVA, and one (25%) developed partial clip detachment at 30 days after the procedure.

There was no statistical difference between patients who developed complications and those who did not regarding their age (75.25 ± 12.42 versus 64.63 ± 9.21, respectively), BSA (1.69 ± 0.11 versus 1.79 ± 0.22, respectively), gender (75% versus 87.5% males respectively), MR etiology (75% versus 50% ischemic, 25% versus 50% non-ischemic), or NYHA pre- or post-mitral clipping. However, the median EuroSCORE was significantly higher in the complicated group (13, IQR= 11.5-14.5) than the non-complicated group (9.5, IQR=8.5-11.5) (Table 5).

**Table 5:** Comparison between non-complicated and complicated groups for demographics

Variables		Not complicated (***N*** = 16)	Complicated (***N*** = 4)	***P*** value
**Age (years)**	Mean ± SD	64.63 ± 9.21	75.25 ± 12.42	0.069
	Range	46-78	57-84	
**Gender**	Female	2 (12.5%)	1 (25%)	0.531
	Male	14 (87.5%)	3 (75%)	
**BSA**	Mean ± SD	1.79 ± 0.22	1.69 ± 0.11	0.370
	Range	1.55-2.3	1.55-1.78	
**MR etiology**	Ischemic	8 (50%)	3 (75%)	0.730
	Non-Ischemic	8 (50%)	1 (25%)	
**NYHA class** **Pre**	I	0 (0%)	0 (0%)	0.197
	II	0 (0%)	0 (0%)	
	III	13 (81.2%)	2 (50%)	
	IV	3 (18.8%)	2 (50%)	
**NYHA class** **Post**	I	12 (75%)	2 (50%)	0.329
	II	4 (25%)	2 (50%)	
	III	0 (0%)	0 (0%)	
	IV	0 (0%)	0 (0%)	
**EuroSCORE**	Median (IQR)	9.5 (8.5-11.5)	13 (11.5-14.5)	0.028*
	Range	7-13	11-15	

*N* Number, *SD* Standard deviation, *IQR* Interquartile range, *BSA* Body surface area, *MR* Mitral regurgitation, *NYHA* New York Heart Association, *EuroSCORE* European System for Cardiac Operative Risk Evaluation

^*^Significant

Follow up of the patients’ over 6 and 12 months revealed significant improvement of the MR grade that started at 1 month and continued at 6 months as well as at 1 year following the MV clipping. There was significant decrease of the median MR grade at each of 1 month, 6 months, and 1 year following the mitral valve clipping in comparison to the median base line MR grade (*P*1, *P*2, and *P*3 <0.001). The same was observed for NYHA class where patients showed significant improvement from the first month of the follow-up and continued along the first year where patients showed significantly lower median NYHA class at 1 month (1.0, IQR = 1.0-2.0), at 6 months (1.0, IQR=1.0-1.5), and at 1 year (1.0, IQR=1.0-1.0) than the median base line NYHA class (3.0, IQR=3.0-3.5), *P*<0.0001. Furthermore, the mean EF was significantly higher at 1 month (58.8 ±8.4, *P*1=0.002), 6 months (62.0 ±6.6, *P*2<0.001), and at 1 year (62.8± 6.2, *P*3<0.001) than the recorded mean EF before the mitral valve clipping (53.8 ±7.8). Alternatively, there was no significant improvement in LA neither at 1 month nor at the 6 months or 1 year following the mitral valve clipping; the mean LA showed non-significant differences at the different times of follow-up (*P*=0.307) (Table 6).

**Table 6:** Follow up of the mitral regurgitation grade, NYHA class, ejection fraction, and the left atrium

		Pre-operative	1 month	6 months	1 year	Test statistic	***P*** value	Post hoc analysis	
MR grade	Median (IQR)	4.0 (4.0-4.0)	2.0 (1.5-2.0)	2.0 (2.0-2.0)	2.0 (2.0-2.0)	54.190	<0.001*	*P*1<0.001^a^ *P*2<0.001 ^a^ *P*3<0.001 ^a^	*P*4=0.083 *P*5=0.083 *P*6>0.999
**NYHA class**	Median (IQR)	3.0 (3.0-3.5)	1.0 (1.0-2.0)	1.0 (1.0-1.5)	1.0 (1.0-1.0)	56.302	<0.001*	*P*1<0.001 ^a^ *P*2<0.001 ^a^ *P*3<0.001 ^a^	*P*4=0.157 *P*5=0.083 *P*6=0.317
**EF**	Mean± SD	53.8 ±7.8	58.8 ±8.4	62.0 ±6.6	62.8± 6.2	22.872	<0.001*	*P*1=0.002 ^a^ *P*2<0.001 ^a^ *P*3<0.001 ^a^	*P*4=0.104 *P*5=0.024 *P*6=0.279
**LA**	Mean± SD	44.9±3.2	43±2.2	43±2.3	43±2.1	2.8	0.04	*P*1=0.009 *P*2=0.011 *P*3=0.011	*P*4=1.0 *P*5=0.88 *P*6=0.88

*P1* Pre versus 1 month, *P2* Pre versus 6 months, *P3* Pre versus 1 year, *P4* 1 Month versus 6 months, *P5* 1 Month versus 1 year, *P6* 6 Months versus 1 year, *SD* Standard deviation, *MR* Mitral regurgitation, *EF* Ejection fraction, *LA* Left atrium, *NYHA* New York Heart Association

*P*^a^ was adjusted for multiple comparisons (Bonferroni correction) resulting in a significant level at *P*<0.008

*Significant at *P* <0.05

## Discussion

Edge-to-edge mitral valve repair can be done by a less invasive percutaneous implantation of a clip (MitraClip) that grasps and approximates the edges of the mitral leaflets at the regurgitant jet orifice. This technology was developed in an attempt to imitate the surgical approach for mitral repair, which involves approximation of the mitral leaflets with a suture to create a double orifice [11]. Our study describes and analyze the Egyptian very first experience using the MitraClip in repairing significant MR. We intended to evaluate this therapy in patients having grades 3-4 MR with high surgical risk. Most of our patients presented with impaired LV function or high burden of comorbidities.

### Baseline clinical characteristics

Regarding demographic data, NYHA class and EuroSCORE, the study results were in line with Tamburino et al. [7] who reported that 31 patients were involved in their study [age 71 (IQR 62-79) years, male 81%].

Fifty eight percent (18 of our patients) presented with functional disease and 13 patients (42%) presented with organic degenerative disease. Among patients with functional MR, 67% had a previous old history of coronary artery disease.

Moreover, Sürder et al. [12] reported that the median age of their patients was 77 years, 67% of them were males, 62% of them were NYHA III, and 20% of them were NYHA IV. Ailawadi et al. [13] found that 62% of the studied patients were NYHA III and 21% of them were NYHA IV. However, Gaemperli et al. [14] found that the mean age of their patients was 78 years and 58% of them were males. The type of MR was degenerative in 16 (48%), functional in 15 (45%), and mixed in 2 (6%) patients.

The present study showed that patients varied for their HAS-BLED score with a median value of 1 (range, 0-4). Coronary angiography showed that 35% of our patients had previous PCI, while 45% had normal coronary anatomy. These results were supported by Feldman et al. [8] who reported that among patients with functional MR, there was a history of coronary artery disease in 74% and previous bypass surgery in 43%.

### Procedure characteristics

In the current study, patients had significant improvements in their NYHA class and echocardiographic assessments following MV clipping. These results were supported by Khamis et al. [11] who reported an improvement in the MR severity in all patients as assessed acutely after MV repair by MitraClip system. Moreover, Whitlow et al. [15] reported that the MitraClip device reduced MR in the majority of patients deemed at high risk of surgery, resulting in an improvement in clinical symptoms and significant LV reverse remodeling over 12 months.

Additionally, Herrmann et al. [16] reported acute procedural success, safety, and 1-year efficacy with MitraClip therapy similar for patients with and without AF.

Furthermore, Armstrong et al. [17] concluded that subjects with thicker anterior mitral leaflets and more significant mitral incompetence were more likely to receive 2 MitraClip devices. Immediate and long-term reductions in MR were similar regardless of the number of devices implanted at the time of the procedure. According to Foster et al. [18], patients with pre-existing LV dysfunction demonstrated reverse remodeling and improved LV ejection fraction at 12 months after percutaneous MV repair with the MitraClip device. Furthermore, Gonzalez et al. [19] stated that MitraClip has become available as a treatment option for MR in high-risk surgical patients as it has been showing a high safety profile and a good middle-term effectiveness performance.

### Clinical outcome and follow-up

Our study has demonstrated that MitraClip device therapy is reliable, applicable, and effective, with procedural success achieved in almost all patients (100%). There was no procedural mortality or MAE at 30 days. Successful placement of the MitraClip device was associated with a reduction in MR severity by ≥ 2 grades in all patients. The present study showed significant improvements in patients’ right-side heart failure symptoms and their laboratory values following MV clipping. However, AF status did not change significantly. These results were in line with Tamburino et al. [7] who reported an improvement of clinical symptoms in all patients after percutaneous MV repair with the MitraClip system. Furthermore, Ailawadi et al. [13] concluded that transcatheter MV repair with the MitraClip in patients with secondary MR was associated with acceptable safety, reduction of MR severity, symptom improvement, and positive ventricular remodeling.

In the present study, four (20%) patients developed complications. These included two cases of bleeding that needed blood transfusion, one case of ischemic CVA, and another one who developed partial clip detachment at 30 days after the procedure.

The current study showed that there was no significant difference between patients who developed complications and those who did not regarding their age, gender, BSA, MR etiology, and NYHA pre- or post-mitral clipping. However, EuroSCORE was higher in the complicated group. These results were supported by Hellhammer et al. [20]. They found no statistical significance between diabetic patients who developed complications and those who did not with respect to age, gender, and MR etiology after percutaneous MV repair with the MitraClip system.

In the present study, follow-up of the patients at 1 month, 6 months, and at 1 year following mitral valve clipping revealed significant improvement of the MR grade, NYHA class, and EF. This emphasizes 1-year efficacy of the MitraClip system. These results coincide with a systematic review of 16 studies including 2980 patients who underwent MitraClip implantation for moderate to severe MR. It concluded persistent MR reduction in 85.3% of the patients at 30-days follow-up and in 86.9% at a mean follow-up of 310 days (ranging from 80 days to 4 years) [21]. Likewise, follow-up of patients with functional MR who underwent MitraClip implantation showed significant improvement of MR grade, EF, and functional capacity according to NYHA class at 12 months compared to the preoperative values [22]. Moreover, Polimeni et al. have recently concluded that LVEDVi and NYHA class are predictors of rehospitalization for heart failure or cardiovascular death in patients having MitraClip system. Thus, the continued effective MR reduction with improved NYHA class for 1 year in our study could increase the survival and reduce the rehospitalization rate in MR patients [23].

### Limitations of the study

Small sample of patients included in our non-randomized study was a main limitation. In addition, due to the novelty of the technique, only a long-term follow-up was documented. Larger series and longer follow-up are warranted to determine the safety, efficacy, and durability of the MitraClip system, enabling further investigation on different patient populations including patients with functional and degenerative MR etiology.

### Clinical implication

Transcatheter edge-to-edge mitral valve repair of severe MR carries high efficacy and safety especially at high-risk patients for surgical mitral valve replacement.

## Conclusion

Transcatheter MV repair with the MitraClip device in patients with severe MR was associated with a favorable safety profile and better clinical outcomes, including positive ventricular remodeling, improved symptoms, and stable MR reduction.

## Data Availability

Our retrospective cross-sectional study data used to support the findings of this study are available from the corresponding author upon request.

## Abbreviations

ACC: American college of cardiology
AF: Atrial fibrillation
AHA: American heart association
ASE: American society of echocardiography
BSA: Body surface area
EVEREST: Endovascular valve edge-to-edge repair study
ICH: International council for harmonization
IQR: Inter-quartile range
LA: Left atrium
LVED: Left ventricular end diastolic diameter
LVES: Left ventricular end systolic diameter
MR: Mitral valve regurgitation
MV: Mitral valve
NYHA: New York heart association
PISA: Proximal isovelocity surface area
Pro-BNP: Pro-brain natriuretic peptide
TAPSE: Tricuspid annular plane systolic excursion
TEE: Trans-esophageal echocardiography
TTE: Transthoracic echocardiography
